# Patient Simulation: A Literary Synthesis of Assessment Tools in Anesthesiology

**DOI:** 10.3352/jeehp.2009.6.3

**Published:** 2009-12-20

**Authors:** Alice A. Edler, Ruth G. Fanning, Michael. I. Chen, Rebecca Claure, Dondee Almazan, Brain Struyk, Samuel C. Seiden

**Affiliations:** 1 Department of Graduate Medical Education, Stanford Hospitals and Clinics, Stanford, CA.; 2 Department of Anesthesia, Stanford University School of Medicine, Stanford, CA.; 3 Department of Anesthesia, Children's Hospital of Philadelphia, Philadelphia, PA, USA.

**Keywords:** High-Fidelity Patient Simulation, Anesthesiology, Patient Simulation, Performance Assessment, Systemic Review, Test Theory

## Abstract

High-fidelity patient simulation (HFPS) has been hypothesized as a modality for assessing competency of knowledge and skill in patient simulation, but uniform methods for HFPS performance assessment (PA) have not yet been completely achieved. Anesthesiology as a field founded the HFPS discipline and also leads in its PA. This project reviews the types, quality, and designated purpose of HFPS PA tools in anesthesiology. We used the systematic review method and systematically reviewed anesthesiology literature referenced in PubMed to assess the quality and reliability of available PA tools in HFPS. Of 412 articles identified, 50 met our inclusion criteria. Seventy seven percent of studies have been published since 2000; more recent studies demonstrated higher quality. Investigators reported a variety of test construction and validation methods. The most commonly reported test construction methods included "modified Delphi Techniques" for item selection, reliability measurement using inter-rater agreement, and intra-class correlations between test items or subtests. Modern test theory, in particular generalizability theory, was used in nine (18%) of studies. Test score validity has been addressed in multiple investigations and shown a significant improvement in reporting accuracy. However the assessment of predicative has been low across the majority of studies. Usability and practicality of testing occasions and tools was only anecdotally reported. To more completely comply with the gold standards for PA design, both shared experience of experts and recognition of test construction standards, including reliability and validity measurements, instrument piloting, rater training, and explicit identification of the purpose and proposed use of the assessment tool, are required.

## INTRODUCTION

"Whatever exists at all exists in some amount. To know it thoroughly involves knowing its quantity as well as its quality" Thorndike EL [1].

Professional education, medicine included, recognizes that for an expert, patient/client-centered practice, competencies beyond purely fact-based knowledge and technical skills are required [2, 3]. These competencies span multiple professional fields and include communication, clinical reasoning and decision making, and reflection in daily practice.

The medical education profession has formalized the combinations of knowledge, skills and attitudes (KSA) into six constructs, medical knowledge, patient care, communication and interpersonal skills, professionalism, practice based learning and improvement, and systems based medicine [4]. However, medical educators are now struggling with methods of authentic assessment for not only those factual knowledge and technical skills but also for the more psychologically based constructs of communication, life long learning and interdisciplinary reasoning.

As our concept of occupational competence develops, so must our assessment tools; beyond pencil and paper tests of knowledge recall, to include higher level cognitive and behavioral assessments [5]. Such assessments require challenging educational techniques for teaching and sophisticated psychometric methods for assessment. Medical education, in particular in anesthesiology has taken a lead from the aviation industry and now is an avid consumer of multiple forms of patient simulation-based teaching and performance assessment (PA), such as high-fidelity patient simulation (HFPS) and mixed-modality simulation (HFPS paired with other simulation techniques such as standardized patients), to assess in complex, simulated, life-like healthcare situations. As use of these PA tools increases, the methodologies used for test construction need to be complete and robust.

In this manuscript we have systematically reviewed the current methodological approaches to PA tool construction using HFPS. We have chosen to limit our review to the field of anesthesiology in order to unify our discussion of progress and because, anesthesiology the longest and most productive use of HFPS for PA.

The purpose of our review is as follows: to identify available HFPS PA tools used in anesthesiology, to comment on the quality of each in terms of classic and modern test theory, and to identify areas of needed research and possible means of standardization of test construction methods.

## MATERIALS AND METHODS

### Search strategies

We reviewed the literature to identify studies in which HFPS was used to test performance in anesthesiology practice and education. We used methods of literary synthesis research or systematic non-statistical meta-analysis of research literature described by Bland et al. [6] and Slavin [7]. Literary synthesis is useful in reviews not amenable to statistical meta-analysis, where dependent and independent variables vary from study to study and data are collected using non-statistically compatible instruments.

A professional medical librarian helped design the sensitive search strategy. Subject headings included: "((anaesth*[ti] OR anesth*[it]) AND simulate*[it]) OR (("Anesthesiology" [Mesh]) AND ("Computer Simulation" [Mesh])) OR (("Anesthesiology" [Mesh]) AND ("Patient Simulation" [Mesh] OR "Models, Educational" [Mesh])) OR (("Patient Simulation" [MeSH] OR "Computer Simulation" [MeSH]) AND (anesth* OR anaesth*) AND medical education)." The search was not limited to date of publication, publication type, or language. While preparing this manuscript, we used the "MY MCBI" updating protocol monthly, continuing to collect data through September thirtieth, 2008. Inclusion and exclusion criteria are listed in Table 1.

### Assessment tool analysis

To assess the quality of test construction consistent with recognized test construction methods [8, 9], we analyzed the following:

1) Methods of item selection, including degree of theory-grounded selection of test items, identification of the knowledge domain and skills or behaviors to be assessed.

2) Elements of test construction, including test piloting, rater training, multiple parallel scenarios/testing occasions, the use of varied scoring systems (analytic, holistic or other).

3) Measurement of the score reliability, using reliability indexes from both classic test theory and modern test theory (MTT) methods [10].

4) Degree of appropriateness of the inferences regarding examinees' ability made from these scores. Based on reported conclusions and stated validity claims in terms of standard psychometric definitions of content, criteria, and construct validity.

5) Practicability and usability of tools (Table 2).

### Data extraction and analysis

AE and RF (the initials refer to the authors) reviewed the abstracts for all citations and identified manuscripts for full review if they matched the inclusion criteria listed in Table 1. Then a research assistant reviewed the manuscripts' bibliographies and crosschecked with the original search to identify additional citations not found in the initial search. For abstracts of unpublished research, the primary author was contacted and asked for a copy of the manuscript.

AE and RF reviewed and coded all selected manuscripts. Four secondary reviewers (MC, DA, BS, and RC) again reviewed and confirmed the findings of methods of item selection, elements of test construction, and type of statistical reliability measures. Secondary raters received instruction on statistical and qualitative methods of analysis. Any further disagreements were resolved by consensus among the primary and secondary reviewers.

### Statistical analysis

We used SPSS (SPSS Inc., Chicago, IL, USA) to obtain descriptive statistics and Coefficients of Correlation for agreement between primary and secondary reviewers.

## RESULTS

Investigators retrieved 412 articles, whose abstracts they and other members of the Department of Anesthesia at Stanford School of Medicine translated from Danish, German, Italian, and Japanese into English. Fifty studies met the inclusion criteria [11-60]. The Coefficient of Correlation for rater agreement between primary and secondary rater in the initial comparison was 0.76.

### Methods of item selection

**Fig. 1:**
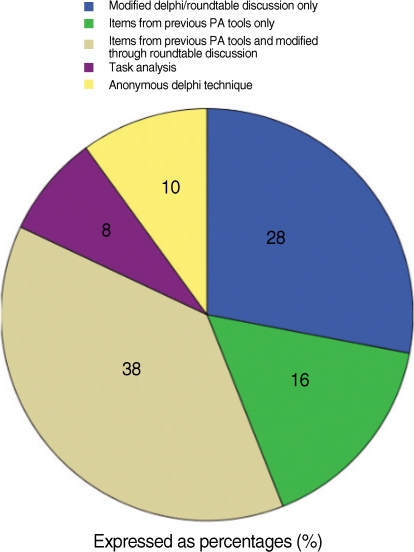
Methods of item selection.

In our review, item selection methods varied considerably, including: round-table discussion, reported as modified Delphi techniques; task analysis; formal Delphi consensus of expert opinion, and internal consistency with items shown to produce reliably scores on previous tests. The most commonly reported item-selection method was round-table discussion among test designers, frequently termed "modified Delphi technique" coupled with items from previous published PA tools. Thirty eight percent of the studied reported this method for item selections. In sixteen percent of the studies, items were chosen only from previously published HFPS assessment tools with out modification of items and twenty eight percent came from exclusively from roundtable discussion among text designers. Ten percent used anonymous Delphi methods for item identification. Eight percent of the studies used formal Task Analysis. However, a valuable tool for item selection and refinement, item response theory (IRT), was not reported in any of these investigations. IRT allows test designers to determine the relative difficulty of test items, completeness of KSA domain sampling. Both of which are critical to the use of any PA for determination of minimal competency standards for credentialing (Fig. 1).

### Elements of test construction

**Fig. 2:**
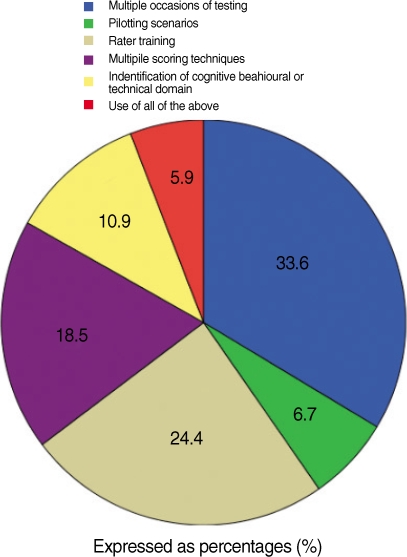
Test refinement methods.

Performance assessments, which include multiple subtests, provide more information about the examinees true ability than those testing situations in which only one assessment is obtained. In forty percent of the studies multiple scenario/occasions of testing were the only method of item refinement used (Fig. 2).

Twenty eight percent of the studies reported rater training prior to actual scoring; Twenty-one studies used multimodal scoring techniques, of which the most common of which were analytic checklists or holistic rubrics for performance. Only fourteen percent of the investigators used scenario piloting to identify problems within the scenario itself.

Many of the investigators, twenty eight percent, used 2 or more methods for item refinement. The most common combination of techniques was multiple scenarios with rater training. However only seven percent used all of the above methods listed.

### Measurement of the score reliability

**Fig. 3:**
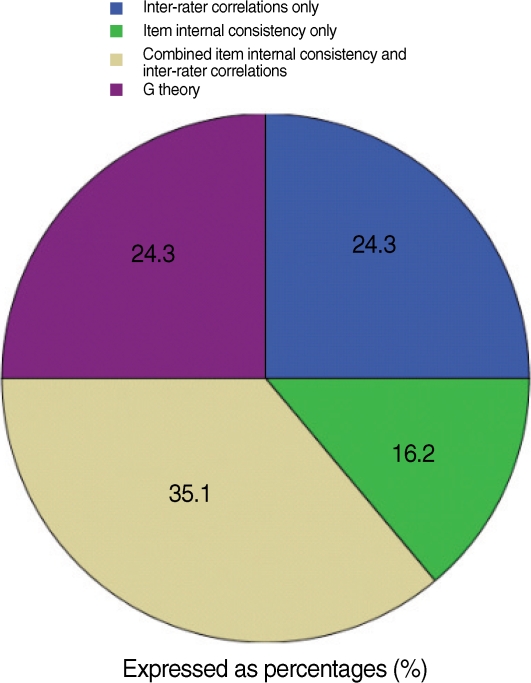
Score reliability measures.

Inter-rater agreement as the sole measure of reliability was noted in twenty four percent of the studies; most of these studies were preformed prior to the year 2000. A variety of rater agreement statistics were used. The most common was intra-class correlations for variance estimations. Other methods used included Kappa for rater agreement, Pearson's correlation, and simple percentage agreement. The degree of rater agreement varied moderately between studies (0.56-0.99). However, not all studies documented rater training, and it was not possible to examine the relationship between rater training and subsequent rater agreement statistics (Fig. 3).

Thirty five percent of the studies reported using measures of internal consistency of items or subtests along with interrater agreement. When we examined the methods used to analyze score reliability from individual items, again, the most common estimation was intra-class correlations. For the most part, these reliability results were moderate.

Only sixteen percent of the studies used MTT, in particular generalizability theory (G theory), to examine the relative internal consistency of items, including the interactions between raters, occasions of testing, and/or other covariates. G theory, unlike correlationally-based intra class coefficient (ICC) or Kappa, derives from analysis of variance, and it can statistically describe the individual components of score error that arise separately from the examinee, the raters, the test items or any number of other confounding conditions that may contribute to score error. Within the last reviewed year, 2007-2008, all published studies describing score reliability estimations have included G theory, vastly improving our ability to discriminate sources of error and assure that differences in scores are truly from differences in examinees' abilities.

An even greater value of G theory is factor analysis, which allows a second type of analysis, decision-making studies (D study). D studies estimate the reliability of the score if any of the sources of that score error are changed. For example, an increase or decrease in the number of raters, occasions of testing and so forth. D studies allow test designers to assure acceptable reliability measures prior to testing rather than post hoc as with ICC. However, only two studies used G theory decision-making analysis (D studies) to maximize the reproducibility of their scoring systems pre-testing.

For the most part over the years in which the studies were performed, we noted a progressive improvement of score-reliability measurements, including the use of combined rater and internal item consistency statistics or the use of analysis of score variance through Generalizability statistics.

### Degree of appropriateness of the inferences regarding examinees' ability made from these scores

The second essential consideration for evaluating the quality of an assessment tool is validity; an attribute of the inferences about the examinees derived from the test scores and not the test, itself. Tests produce valid scores if the inferences about the examinee's ability made from those scores are correct. Though definitions of validity are currently evolving, for the purposes of this review, the authors will use classic validity definitions of criteria, content, or construct [61].

Early studies were limited to comments on *face-validity* conclusions, more recent investigators have reported *content*, *criteria*, and construct validity conclusions [10]. The most common method used to assess content validity, the adequate and complete representation of subject-matter content in test items, was expert opinion through round-table discussion or "modified Delphi technique". As described "modified Delphi methods" only roundtable discussion of items. Several investigators attempted to demonstrate content validity by comparing their tests with previously identified subject-matter-based tests used. On the whole, PA tools whose resultant scores were compared with only paper and pencil test scores faired poorly [35, 40, 48]. Only when a broader view of competency was taken to include both higher-level cognitive skills and technical skills [43-45], the agreement was improved.

Criteria validity, either concurrent or predictive, is the ability of the resultant scores to correspond with scores from other recognized assessments of similar KSA's. Criteria validity is used to assure that inferences about the abilities that the examinee currently demonstrates or will demonstrate are correct. Investigators in this review, most frequently reported concurrent criteria validity, matching simulation-based assessment scores with level of clinical anesthesia training. The results of these correlations were moderate to strong for criteria-based inferences [15, 20, 33, 42, 43, 45, 49, 50, 52]. This ability of simulation-based assessment to discriminate between levels of training was demonstrated but limited. Most simulation-based assessment scores could distinguish between early trainees and academic faculty, and some could distinguish between levels of anesthesia training or other professional anesthesia providers but not consistently. None of the reviewed assessment tools reported item difficulty indexes.

Construct validity of score inferences is the most difficult to conceptualize and assess; currently the very concept of construct validity is under question. Cronbach and Meehl [61] defines construct validity as "the ability to infer correct qualities [sic, of the examinee] which are not operationally defined". The difficulty lies in the fact that some constructs, such as teamwork, communication, or professionalism, are strongly influenced by culture, gender, or professional identity and cannot be easily and universally operationalized. Fletcher et al. [24] and Weller [58] have provided excellent models of construct validation in behaviorally-based assessment tools. In these studies, elements of teamwork were identified by task analysis and then examined statistically through factor analysis to see if each item correlated with others and with the test as a whole. All estimations of the final scoring systems displayed good to excellent psychometric qualities.

The final two quality characteristics of quality test construction-practicality and usability-are external to the tool itself but juxtaposed [62, 63]. Though many studies reported likeability of instruments, the use of scenario piloting, rater training and multiple question formats to improve usability was not consistent. The information on the practicality of HFPS in was limited and contained in editorial comments about the cost/demands of an HPFS center and found principally in the non-anesthesiology literature [64-68]. We found no explicit cost/benefit literature in anesthesiology.

Finally tests are designed for a purpose, to identify areas of needed improvement (formative tests) or to assess minimally acceptable competence (summative tests). We noted that manuscripts published between 2007 and 2008, substantially improved in reporting the purpose of the test. Two excellent examples are use of HFPS PA as a method to improve the curriculum [59] and the use of HFPS testing for the determination of minimal competence or "cut scores" for summative assessment of examinees' ability [16].

## DISCUSSION

We have found progressive and noteworthy improvement in quality of performance testing using anesthesiology-based HFPS over the past two decades with dramatic increases in the quality of item selection and test construction in the published tools since 2007. Since 2007, there appears to be a more universal acceptance of standard PA tools construction methods. Techniques for careful item selection and minimization of bias through piloting, rater training and multiple subtests/scenarios are also improving but inconsistently. For example, Task Analysis remains the gold standard for identifying skills and attitudes. However few of the studies employed Task Analysis for item selection [25, 30, 48, 54].

Likewise score reliability measures are improving, but the relatively weak measures of internal consistency when comparing scores across varied subtest/scenarios raises the question if examiners are choosing scenarios, which assess the same KSA across these varied subtest/scenarios. As an example, KSA needed for the correct management of ventilator settings in the patient with lung disease are not necessarily the same KSA needed for management of team efforts in the acute treatment of trauma from motor accident. This maybe the cause of lower correlations between scores on differing subtest/scenarios as seen in several of the studies. A more careful look is needed to identify testing scenarios that contain equivalent KSA's assessments. Here the introduction of MTT and IRT maybe of great help.

The recent literature shows a greater use of G theory for reliability estimates but still a severe underuse of D study estimations. D study estimations, though not statically a sample size estimation but conceptually similar, essentially improve the "bang for the buck" when choosing the number of raters, items, testing scenarios etc. A very important feature in these labor and cost intensive HFPS performance assessments.

In another encouraging work, investigators in Israel found reliable inter rater agreement between US and Israeli raters when using the same scenario set. This finding suggests that with proper PA construction and the use of decision-making studies to minimize score error, scenarios and PA tools may be shared in similar practice venues; a particularly pertinent point, as the construction of highly reliable and valid assessment tools that are practical to develop and cost efficient to implement. Developing such tests/tools that are universally applicable and shareable throughout the medical community will be invaluable.

Validity issues still plague the HFPS performance assessment literature. Several studies do demonstrate concurrent validity but only at a gross level, novice verses experienced practitioner. The reasons for this are unclear, however may include incomplete domain sampling. Other possible explanations include inappropriate scaling, low discrimination indexes of the items, or non-linear average item difficulty.

One fascinating point, which bridges both criteria and construct validity issues, was the finding by Devitt et al. [22] that simulation based performance assessment can differentiate between academic anesthesia trainees and their faculty but not between faculty and their private practitioners counterparts with equal levels of practice experience. This raises the question whether different sets of KSA are needed for academic anesthesia practice where trainees are present as compared with practices in which trainees are not. And the more profound question: if performance assessment tools used for credentialing are created by academic anesthesiologists, are the scores obtained from these tools equally valid for non-academic anesthesiologists? This perhaps is one of the most important issues to address prior to the use of simulation based PA in professional credentialing.

The exponential growth of HFPS for PA in anesthesiology during the past two decades has resulted in greater expertise in both test construction and execution. However, although the quality of PA tools has dramatically improved, they need further refinement. For example, test-construction methods, rater training, and piloting and scripting of scenarios should be standardized and uniformly applied. Combined scoring systems should address not only a checklist of technical skills but also global latent trait measurement. In addition, MTT could diminish sources of error variance and gaps in item discrimination ability. The relevance of educational and assessment methods has moved beyond the realm of medical educationalists into the realm of mainstream practice. As we all face assessment of competence in dynamic environments over the next few decades, it is pertinent that we ensure the validity and reliability of test scores to adequately reflect examinees' true competence in the practice of medicine.

## CONFLICT OF INTEREST AND DISCLAIMERS

There is no conflict of interest, no disclaimers and no financial support recordable.

## Figures and Tables

**Table 1:**
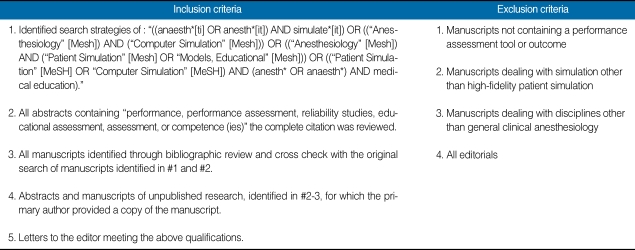
Inclusion and exclusion criteria

**Table 2:**
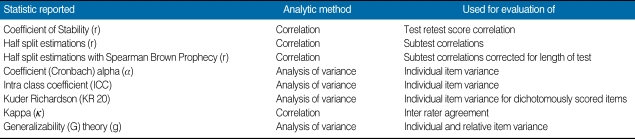
Reliability estimations: methods of agreement/reliability estimations and their uses
